# HLA-E expression in cervical adenocarcinomas: association with improved long-term survival

**DOI:** 10.1186/1479-5876-10-184

**Published:** 2012-09-04

**Authors:** Vivian M Spaans, Alexander AW Peters, Gert Jan Fleuren, Ekaterina S Jordanova

**Affiliations:** 1 Department of Pathology, Leiden University Medical Center, PO Box 9600, 2300, RC, Leiden, the Netherlands; 2 Department of Obstetrics and Gynaecology, Leiden University Medical Center, PO Box 9600, 2300, RC, Leiden, the Netherlands

**Keywords:** HLA-E, Immune surveillance, Immune escape, Cervical cancer, Cervical adenocarcinoma, Cervical adenosquamous carcinoma, Cervical squamous cell carcinoma

## Abstract

**Background:**

Cervical cancer is the third most common cancer in women worldwide. The most common histopathological subtype is cervical squamous cell carcinoma (SCC, 75-80%), followed by adenocarcinoma (AC) and adenosquamous carcinoma (ASC; together 15-20%). Rising incidence rates of AC have been observed relative and absolute to SCC and evidence is accumulating that cervical AC is a distinct clinical entity. Cervical SCC, ASC, and AC are caused by a persistent infection with high-risk human papillomavirus (HPV) and failed control of the immune system plays a pivotal role in the carcinogenesis of all three histopathological subtypes. Human leukocyte antigen E (HLA-E), a non-classical HLA class Ib molecule, plays an important role in immune surveillance and immune escape of virally infected cells. In this study we investigated HLA-E expression in three well-defined cohorts of cervical AC, ASC, and SCC patients, and determined whether HLA-E expression was associated with histopathological parameters and patient survival.

**Methods and results:**

HLA-E expression was assessed by immunohistochemistry on formalin-fixed, paraffin-embedded tissue sections of 79 SCC, 38 ASC, and 75 AC patients. All patients included were International Federation of Gynaecology and Obstetrics stage I-II and underwent radical hysterectomy with lymphadenectomy as primary treatment. Significant differences between the histopathological subgroups were detected for age distribution, HPV positivity, HPV type distribution, tumour size, tumour infiltration depth, lymph-vascular space invasion, and adjuvant radiotherapy. High expression of HLA-E was found in 107/192 (56%) cervical carcinomas, with significantly more overexpression in cervical AC compared to SCC and ASC (37/79 SCC, 18/38 ASC, and 52/75 AC; *P* = 0.010). High HLA-E expression in cervical AC was associated with favourable long term disease-specific and recurrence-free survival (*P* = 0.005 and *P* = 0.001, respectively).

**Conclusion:**

High expression of HLA-E occurred in the majority of all histopathological subtypes of cervical cancer; especially in cervical AC. High HLA-E expression in cervical AC was associated with improved patient survival. This study also highlights the importance of careful evaluation of cervical carcinomas to distinguish histopathological subtypes. In the future, insight into the biological behaviour and distinct molecular carcinogenetic processes of the AC, ASC, and SCC subtypes may contribute to the development of more tumour-specific treatment strategies.

## Background

Cervical cancer is the third most common cancer and fourth leading cause of cancer death in women worldwide [1]. Invasive carcinomas of the cervix are classified into subtypes based on their histological features. Cervical squamous cell carcinoma (SCC) comprises approximately 75-80% of all cervical carcinomas and is the most common histological variant, followed by cervical adenocarcinoma (AC) and adenosquamous carcinoma (ASC), together comprising approximately 15-20% of all cervical carcinomas [2-4]. The overall incidence of invasive cervical cancer, particularly that of SCC, has declined in developed countries since the introduction of cytological screening programmes that have improved detection of premalignant stages. In contrast, the absolute and relative incidence rates of AC and ASC have been stable or have even increased in some countries over the last two decades. This trend was observed predominantly in younger women and in developed countries [5-10].

The rising incidence of AC has resulted in a heightened interest in this tumour subtype. AC differs from ASC and from SCC not only in histological features, but also in biological behaviour, patterns of tumour growth, metastasis, and sensitivity to chemo- and radiotherapy [11,12]. Furthermore, compared to SCC, AC and ASC are associated with worse prognosis and survival, with higher recurrence rates, and with different responses to similar treatment strategies [10,11].

Concerning aetiology, AC, ASC, and SCC have one foremost aspect in common: they are caused by a persistent cervical infection with high-risk human papillomavirus (HPV) [13-15]. The immune system plays a pivotal role in cervical carcinogenesis. Although most HPV infections are transient and efficiently cleared by the immune system, a small percentage of infected women develop premalignant lesions and eventually invasive cervical cancer. The progression from HPV infection to persistent infection to invasive cervical cancer is largely determined by failed control of the immune system and by the ingenuity of the infected (tumour) cells in escaping the host’s immune surveillance [16,17].

Human leukocyte antigen E (HLA-E) is a non-classical major histocompatibility complex class I molecule [18]. HLA-E can be expressed on the cell surface of nucleated cells and presents a limited variety of peptides to cytotoxic T lymphocytes (CTLs) and natural killer (NK) cells. Compared to the classical HLA class I molecules (HLA-A, -B, -C), HLA-E is almost non-polymorphic and has low cell surface expression in normal tissues [18]. Upregulation of HLA-E on the cell surface and consequent recognition by CTLs and NKs lead to either an inhibitory or a stimulatory response, depending on subsequent receptor binding and the intended cytotoxic response [18]. Binding to the CD94/NKG2A receptor leads to an inhibitory response, whereas binding to the CD94/NKG2C receptor leads to a stimulatory response. The first large studies concerning the function of HLA-E addressed maternal-foetal interaction in pregnancy. Expressed on placental tissue, HLA-E plays an important role in pregnancy and enables the mother’s immune system to accept the foetus, which would otherwise be detected as “foreign” [19]. Additional studies provided evidence that HLA-E expression contributes to immune surveillance and/or immune escape of virally infected cells, stressed cells, and tumour cells [18,20-22].

HLA-E is only weakly expressed in normal cervical epithelium [23]. HLA-E expression was found to increase significantly with the progression from normal epithelium to virally infected lesions to cervical intraepithelial neoplasia grade 1, grade 2–3, and cervical cancer [24]. In 83% of cervical cancer samples, HLA-E expression was higher in tumour cells compared to the paired normal cervical epithelium [23]. Gooden et al. analysed 149 cervical carcinoma patients for HLA-E expression [23]; and although the research did not focus on analysis of differences by histopathological subtypes, only 4/26 (15%) AC/ASC cases exhibited low expression of HLA-E compared to 29/123 (24%) of the SCC cases. This difference in HLA-E expression between the histological subtypes was remarkable and motivated investigation of the role of HLA-E in the AC and ASC subtypes of cervical cancer.

Given the rising incidence rates of cervical AC, it is important to further distinguish the biological behaviour and molecular carcinogenesis of AC compared to SCC and ASC. Insight in these processes further defines cervical AC as a distinct clinical entity and may lead to the design of more tumour-specific treatment strategies to fight this specific tumour subtype.

Here, for the first time, we report the expression of HLA-E in a large and well-defined cohort of cervical AC patients and compare this expression with our previous measurements of HLA-E expression in cervical SCC and ASC. Furthermore, we investigate the associations between HLA-E expression, clinicopathological parameters, and disease-specific and recurrence-free survival of these cervical cancer patients. In this well-defined cohort of cervical AC patients, HLA-E is more frequently overexpressed than in cervical SCC and ASC. High expression of HLA-E in AC patients was found to be associated with improved long-term disease-specific and recurrence-free survival.

## Methods

### Patients

This study included a total of 192 women who were diagnosed with primary carcinoma of the uterine cervix. We included all AC and ASC patients and a selected cohort of SCC patients, all FIGO (International Federation of Gynaecology and Obstetrics) stage I-II, who underwent radical hysterectomy with lymphadenectomy as primary treatment between January 1990 and December 2005 at the Leiden University Medical Centre. Patients who had received radiotherapy and/or chemotherapy prior to surgery were excluded. Formalin-fixed, paraffin-embedded tissue blocks containing a representative part of the resected cervical tumour were retrieved from the archives of the Department of Pathology. Conventional histology sections were stained with haematoxylin and eosin and reviewed by a trained pathologist (GJF). Cases with deficient representative tumour material were excluded from further study. Additionally, to carefully discriminate SCC from ASC, conventional histology sections were stained with Periodic Acid Schiff Plus and Alcian Blue (PAS+/AB) and reviewed. Cases with positive staining patterns of intra-cytoplasmic mucus were (re)classified as ASC. The following rare histological variants of cervical carcinoma were excluded because their relation with HPV infection is less well established: clear cell AC (N = 6), serous AC (N = 2), mesonephric AC (N = 2), minimal deviation AC (N = 4), glassy cell carcinoma variant (N = 4), adenoid carcinoma (N = 1), neuroendocrine carcinoma (N = 2), undifferentiated carcinoma (N = 2), mesenchymal carcinoma (N = 1), and mixed carcinoma (N = 9). The final study cohort consisted of 75 AC cases, 35 ASC cases and 79 SCC cases. This distribution of tumour subtypes is not a reflection of the population distribution (80% SCC versus 20% AC/ASC); however, as we were especially interested in the cervical AC and ASC subtypes, a smaller SCC cohort was considered sufficient for valid comparisons.

Clinical charts and the original pathology reports for all 192 patients were reviewed and the following data were collected: age of the patient at date of primary treatment (surgery), FIGO stage, tumour size, infiltration depth, lymph-vascular space invasion (LVSI), tumour infiltration in the parametria, tumour-positivity of the resection margins, presence of lymph node metastases, and whether the patient had received postoperative radiotherapy. Follow-up data for all patients were collected concerning survival and recurrence of the disease. Disease-specific survival was defined as time in months from date of primary surgery until death by cervical cancer or until date of last follow-up. Recurrence-free survival was defined as time in months from date of primary surgery until date of first recurrence or date of last follow-up in case of no recurrence. All human tissue samples were used in accordance with the guidelines of the Ethical Committee of the Leiden University Medical Center.

### HPV typing

All cervical tumour samples included in this study were typed for HPV as described previously [25]. DNA was extracted from the formalin-fixed, paraffin-embedded tissue blocks. Sections of a paraffin block without tissue were cut between each sample to rule out contamination and to use as a negative control. HPV DNA was amplified using the SPF10 primer set. HPV DNA detection and broad spectrum HPV genotyping were performed using the INNO-LiPA HPV genotyping *Extra* line probe assay (Innogenetics, Ghent, Belgium), which is a highly sensitive hybridisation assay for detecting HPV DNA and specifying HPV genotypes.

### Immunohistochemistry

Clone MEM-E/02 mouse monoclonal antibody against HLA-E (MCA2193, AbD Serotec, Kidlington, UK) was used to determine HLA-E protein expression in tumour cells from all patients included in this study (N = 192). A previously described tissue microarray (TMA) [26] including tissue cores from 15 AC patients and all SCC (N = 79) and ASC (N = 38) patients was used to determine HLA-E protein expression of the tumour cells (partly described previously by Gooden et al [23]). To obtain a high concordance rate with whole tissue slides, only samples with a minimum of two representative tissue cores, both of which contained a minimum of 20% tumour tissue, were used. HLA-E protein expression in all AC patients included in this study (N = 75) was determined on whole tissue sections (4-μm paraffin sections). To verify the comparability of the staining patterns of the TMA and the whole-section stains, 15 AC cases were included on the TMA and were also stained as whole sections; this analysis resulted in highly comparable measurements (mean total score TMA staining 6.13 ± 1.8, mean total score whole section staining 6.00 ± 1.4, *P* = 0.956).

First, the tissue sections were deparaffinised and rehydrated using graded concentrations of ethanol to distilled water; endogenous peroxidise activity was blocked with 0.03% H_2_O_2_/MeOH for 20 minutes. Antigen retrieval was performed in boiling 0.01 M citrate buffer (pH 6.0) for 12 minutes. After two hours of cooling in citrate buffer, slides were washed twice in distilled water and twice in phosphate-buffered saline. Subsequently incubation was performed overnight at room temperature with the primary antibody diluted 1:800 in phosphate-buffered saline containing 1% bovine serum albumin. Second, sections were incubated with BrightVision poly-horseradish peroxidase anti-mouse/rabbit/rat IgG (ImmunoLogic BV, Duiven, the Netherlands) for 30 minutes at room temperature. Washing between incubations was performed three times for five minutes in phosphate-buffered saline. Immune complexes were visualized by applying a 0.05 M Tris–HCl buffer (pH 7.6) containing 0.05% of 3,3’-diamino-benzidine-tetrahydrochloride and 0.0018% H_2_O_2_. After 10 minutes, the reaction was stopped by rinsing with demineralised water. Finally, the tissue sections were counterstained with Mayer’s haematoxylin before addition of a coverslip. Brown cytoplasm and membrane staining of tumour cells indicated positive HLA-E expression. Resident leukocytes and endothelium of local blood vessels were used as internal positive controls, and two extra AC sections stained without primary antibodies were used as negative controls.

### Immunohistochemical evaluation

The staining patterns were scored semi-quantitatively without prior knowledge of clinical and histopathological parameters with the scoring system proposed by Ruiter et al [27]. The percentage of positively stained tumour cells was scored from 0 to 5 to indicate the presence of positively stained tumour cells: absent (<1%, 0), sporadic (1-5%, 1), local (6-25%, 2), occasional (26-50%, 3), majority (51-75%, 4), or large majority (>75%, 5). The staining intensity of positively stained tumour cells was scored from 0 to 3 to reflect negative (0), weak (1), moderate (2), or strong (3) staining intensity. A final score was calculated by totalling the scores for percentage and intensity, resulting in a score from 0 to 8. A final score of 0 indicated negative expression, 2–4 weak expression, 5–6 moderate expression, and 7–8 strong expression.

### Statistics

Statistical analysis was carried out with IBM SPSS Statistics 20.0 for Windows (IBM Corporation, Armonk, NY, USA). Data were processed using the Chi-square test for categorical variables, Student’s t-test for parametric continuous variables, or one-way analysis of variance for numerical data when comparing more than two groups. The Spearman rho correlation coefficient was used to detect correlation in nonparametric data, and the Pearson correlation coefficient was used in cases of normality. Kaplan-Meier survival curves were generated using GraphPad Prism 5.04 (GraphPad Software Inc., LA Jolla, CA, USA) to estimate disease-specific and recurrence-free survival, and the Log Rank test was used to analyse between-group differences in survival. A multivariate analysis was performed using a Cox proportional hazard model with stepwise regression. For all reported tests, *P* values were two-sided and *P* values <0.05 were considered to indicate statistical significance.

## Results

### Patients and survival

A total of 192 patients (median age 44 years, range 24–87 years) were included in this study: 79 (41%) with SCC, 38 (20%) with ASC, and 75 (39%) with AC. Patient and tumour characteristics for the three histological tumour subtypes are presented in Table 1. Significant differences were detected between SCC, ASC, and AC in age distribution, with ASC and AC presenting at relatively younger age (mean age 50, 43 and 45 years, for SCC, ASC and AC respectively; *P* = 0.014). For SCC and ASC, mean tumour size as well as mean tumour infiltration depth were significantly larger than AC, with mean tumour sizes of 37 ± 16 mm, 35 ± 16 mm, and 26 ± 14 mm, respectively (95% confidence intervals (CIs) 34–41 mm, 30–40 mm, and 23–30 mm, respectively; *P* < 0.001) and mean infiltration depths of 15 ± 7 mm, 15 ± 10 mm, and 11 ± 7 mm, respectively (95% CIs 14–17 mm, 11–18 mm and 9–12 mm, respectively; *P* = 0.001).

**Table 1:** Patient and tumour characteristics by histopathological subtypes

	**SCC**	**ASC**	**AC**	***P*value**
	**n = 79**	**n = 38**	**n = 75**	
**Age (years), mean ± sd**	49.8 ± 14.5	43.0 ± 11.7	44.7 ± 13.3	**0.014**
**(95% CI for mean)**	(46.6-53.1)	(39.1-46.8)	(41.6-47.7)	
**FIGO stage I, n (%)**	64 (81)	34 (89)	67 (89)	0.260
**FIGO stage II, n (%)**	15 (19)	4 (11)	8 (11)	
**High-risk HPV positive, n (%)**	75 (95)	37 (97)	64 (85)	**0.036**
**- HPV 16**	51 (68)	19 (51)	28 (44)	**0.003**
**- HPV 18**	13 (17)	12 (32)	28 (44)	
**- Other**	11 (15)	6 (16)	8 (12)	
**Tumour size ≥ 40 mm, n (%)**	31 (41)	16 (42)	11 (16)	**0.001**
**Infiltration depth ≥ 15 mm, n (%)**	39 (51)	13 (34)	17 (24)	**0.003**
**Positive LVSI, n (%)**	47 (60)	19 (53)	23 (38)	**0.037**
**Positive resection margins, n (%)**	22 (28)	4 (11)	17 (23)	0.109
**Positive parametria, n (%)**	8 (10)	4 (11)	4 (5)	0.483
**Positive lymph nodes, n (%)**	27 (34)	9 (24)	15 (20)	0.125
**Adjuvant radiotherapy, n (%)**	48 (61)	18 (47)	30 (40)	**0.034**
**Follow-up time (months), mean ± sd**	199.4 ± 11.9	186.3 ± 16.6	201.9 ± 11.0	0.782
**(95% CI for mean)**	(176.1-222.6)	(153.9-218.8)	(180.3-223.5)	
**Recurrence-free time (months), mean ± sd**	184.2 ± 12.4	168.3 ± 17.6	148.2 ± 10.5	0.977
**(95% CI for mean)**	(160.0-208.4)	(133.8-202.8)	(127.5-168.8)	
**Death, all causes, n (%)**	34 (43)	12 (32)	22 (29)	0.177
**Death by tumour, n (%)**	19 (24)	10 (26)	16 (21)	0.828
**Recurrent disease, n (%)**	21 (27)	10 (26)	19 (24)	0.984

*P* values were calculated using the Pearson Chi-square test for categorical data and one-way analysis of variance for numerical data. Total follow-up time and disease-free time were calculated by the Log Rank (Mantel-Cox) test for the histopathological subgroups separately. *P* values in bold type were considered statistically significant (*P* < 0.05). For nine, six, eighteen, and one cases the tumour size, depth of infiltration, LVSI (lymph-vascular space invasion), and tumour positivity of resection margins, respectively, could not be identified. SCC, cervical squamous cell carcinoma; ASC, cervical adenosquamous carcinoma; AC, cervical adenocarcinoma; FIGO, International Federation of Gynaecology and Obstetrics; HPV, human papillomavirus; CI, confidence interval.

A total of 96/192 (50%) patients were treated with adjuvant radiotherapy. Adjuvant radiotherapy was indicated in cases with tumour-positive lymph nodes (51/192, 27%), tumour infiltration in the parametria (16/192, 8%), tumour-positive resection margins (43/191, 23%), or if two out of three of the following unfavourable prognostic factors were present: LVSI (89/174, 51%), tumour size ≥40 mm (58/183, 32%), and tumour infiltration depth ≥15 mm (69/186, 37%). Follow-up data were collected until December 2011. By that date, the estimated mean disease-specific survival for all patients (independent of histological subtype) was 202 ± 7.4 months (95% CI 188–217 months) and the estimated mean recurrence-free survival for all patients was 183 ± 8.1 months (95% CI 167–198 months). Fifty patients (26%) had suffered from recurrent disease, 45 patients (23%) had died due to cervical cancer, and 23 patients (12%) had died due to other causes. Survival data per histological subtype are presented in Table 1. No significant differences in disease-specific and recurrence-free survival between the histological subtypes were detected by univariate analysis. However, in multivariate Cox regression analysis, histopathological subtype was an independent predictor for disease-specific and recurrence-free survival, as were the presence of lymph node metastasis, tumour size, and tumour-infiltrated parametria. Age, infiltration depth, LVSI, tumour-positive resection margins, postoperative radiotherapy, and FIGO stage were not independent predictors for survival in the multivariate analysis.

### HPV types

All tumour samples included in this study were typed for HPV. High-risk HPV types were detected in 176/192 (92%) patients. HPV 16 and HPV 18 were the most frequent genotypes, identified in 98 (51%) and 53 (28%) patients, respectively. Twenty-five (13%) patients were positive for other high-risk HPV types: eight for HPV 45, four for HPV 52, four for HPV 33, three for HPV 31, two for HPV 68, and four single cases for HPVs 51, 56, 58, and 59. Overall, HPV positivity was more frequently detected in SCC and ASC than in AC (95% and 97% versus 85%, *P* = 0.036). Furthermore, the HPV type distribution was significantly different for all three histological subtypes (*P* = 0.003), with HPV 16 more dominant in SCC than HPV 18 (68% vs. 17%), while HPVs 16 and 18 were detected with equal frequency in AC (44% vs. 44%; Table 1).

### HLA-E (MEM-E/02) expression

**Figure 1:**
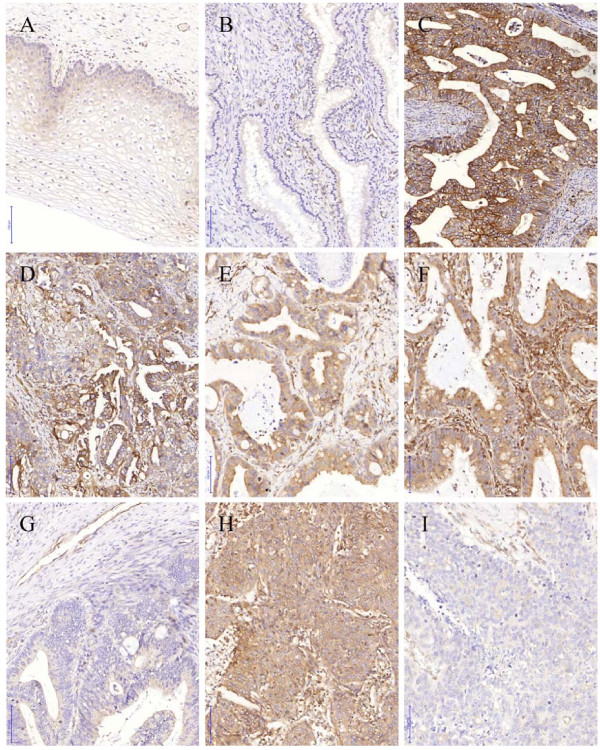
**HLA-E expression in cervical epithelium and cervical carcinoma.** Human leukocyte antigen E (HLA-E) immunohistochemistry was performed with the MEM-E/02 antibody against HLA-E. **A**) Weak HLA-E expression in normal cervical squamous epithelium. **B**) Weak HLA-E expression in normal cervical cylindrical epithelium. **C**) Strong HLA-E expression in endocervical-type cervical adenocarcinoma (AC) **D**) Strong HLA-E expression in endometrioid-type AC. **E**) Moderate HLA-E expression in endocervical-type AC with normal stromal expression. **F**) Moderate HLA-E expression in endocervical-type AC with strong stromal expression. **G**) Weak to negative HLA-E expression in endocervical-type AC. **H**) Strong HLA-E expression in cervical squamous cell carcinoma. **I**) Weak to negative HLA-E expression in cervical squamous cell carcinoma. Bar (blue, left bottom) represents 100 μm.

HLA-E expression was determined in all 192 patients with the HLA-E-specific MEM-E/02 antibody. For 133 cervical cancer patients, HLA-E expression was assessed via tissue cores confined in a TMA; for 74 cervical AC patients, expression was assessed in whole tissue sections (15 AC cases overlap). All negative controls were negative and internal controls (endothelium of local blood vessels and resident leukocytes) were positive for HLA-E. The normal cervical epithelia, when present, were all weakly positive for HLA-E, in accordance with previous reports [23,24] (Figure 1A and B). 

Examples of positive and negative HLA-E expression patterns in cervical carcinomas are depicted in Figure 1 C-I. Staining patterns exhibiting high HLA-E protein expression were detected in the majority of cervical carcinomas. In percentage, 102/192 (53%) carcinomas showed more than 75% of positively stained tumour cells and another 50/192 (26%) carcinomas showed 50-75% of positively stained tumour cells. The intensity of staining varied from weak (in 40%) to moderate (in 34%) to strong (in 15%). Twenty-three (12%) carcinomas scored completely negative, including 12/79 SCC, 9/38 ASC, and 2/75 AC samples (15%, 24%, and 3%, respectively). Sixteen carcinomas (8%) scored only weakly positive (total score 2–4): 5/79 SCC, 1/38 ASC, and 10/75 AC samples (6%, 3%, and 13%, respectively). Sixty-seven (35%) carcinomas scored moderately positive (total score 5–6), including 32/79 SCC, 12/38 ASC, and 23/75 AC cases (41%, 32%, and 31%, respectively). Finally, 86 (45%) carcinomas scored strong positive (total score 7–8), including 30/79 SCC, 16/38 ASC, and 40/75 AC cases (38%, 42%, and 53%, respectively).

To assess whether HLA-E expression was associated with clinical or histopathological parameters, the expression scores were dichotomized based on the median to generate a cut-off score of 6, where a final score of≥6 indicated high HLA-E expression (N = 107, 56%) and a final score of <6 indicated low to negative HLA-E expression (N = 85, 44%). Table 2 contains the associations between HLA-E expression and histopathological parameters. HLA-E expression was significantly higher in cervical AC than in cervical SCC and ASC (*P* = 0.010). In addition, high expression of HLA-E was negatively associated with LVSI (*P* = 0.045).

**Table 2:** Associations between HLE-E expression and histopathological parameters

		**HLA-E low**	**HLA-E high**	***P*value**
		**n (%)**	**n (%)**	
**Histology**	SCC	42 (53)	37 (47)	**0.010**
**(n = 192)**	ASC	20 (53)	18 (47)	
	AC	23 (31)	52 (69)	
**Tumour stage**	FIGO I	72 (44)	93 (56)	0.662
**(n = 192)**	FIGO II	13 (48)	14 (52)	
**HPV**	Negative	7 (44)	9 (56)	0.965
**(n = 192)**	Positive	78 (44)	98 (56)	
**HPV type specific**	HPV 16	42 (43)	56 (57)	0.949
**(n = 151)**	HPV 18	23 (43)	30 (57)	
**Tumour size**	<40 mm	56 (45)	69 (55)	0.997
**(n = 183)**	> = 40 mm	26 (45)	32 (55)	
**Infiltration depth**	<15 mm	47 (40)	70 (60)	0.112
**(n = 186)**	> = 15 mm	36 (52)	33 (48)	
**LVSI**	Negative	32 (38)	53 (62)	**0.045**
**(n = 174)**	Positive	47 (53)	42 (47)	
**Resection margins**	Tumour free	62 (42)	86 (58)	0.178
**(n = 191)**	Tumour positive	23 (54)	20 (47)	
**Parametria involvement**	Tumour free	76 (43)	100 (57)	0.314
**(n = 192)**	Infiltrated	9 (56)	7 (44)	
**Lymph nodes**	Negative	60 (43)	81 (57)	0.426
**(n = 192)**	Positive	25 (49)	26 (51)	
**Adjuvant radiotherapy**	No	37 (39)	59 (61)	0.110
**(n = 192)**	Yes	48 (50)	48 (50)	

Human leukocyte antigen E (HLA-E) expression scores are dichotomized by the median; a final score ≥6 indicates high HLA-E expression, and a final score <6 indicates low to negative HLA-E expression. For nine, six, eighteen, and one cases the tumour size, depth of infiltration, LVSI (lymph-vascular space invasion), and tumour positivity of resection margins, respectively, could not be identified. *P* values were calculated using the Pearson Chi-Square test. *P* values in bold type were considered statistically significant (*P* < 0.05). SCC, cervical squamous cell carcinoma; ASC, cervical adenosquamous carcinoma; AC, cervical adenocarcinoma; FIGO, International Federation of Gynaecology and Obstetrics; HPV, human papillomavirus.

### Association between HLA-E expression and survival

**Figure 2:**
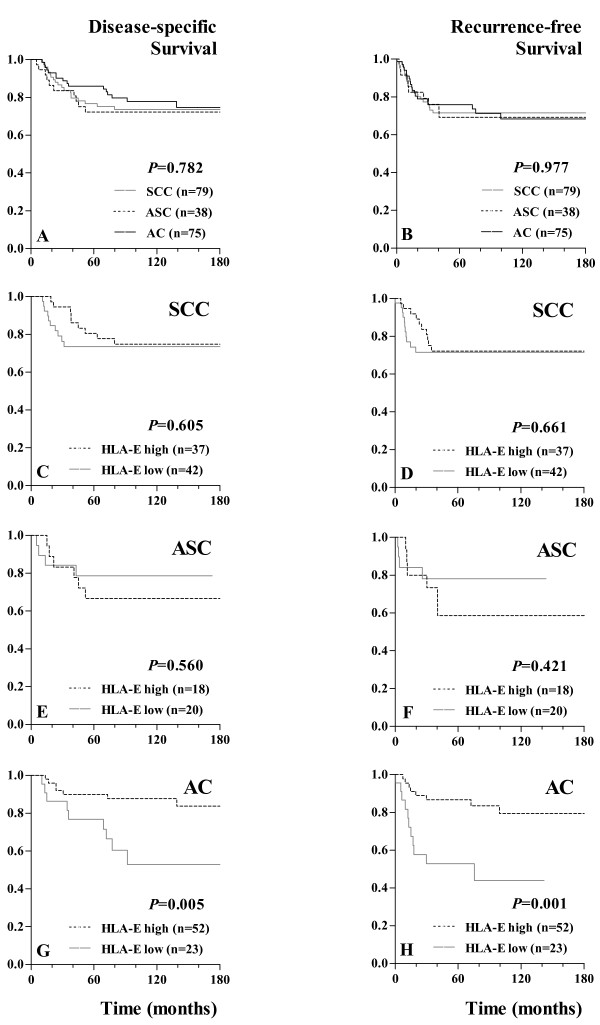
**Disease-specific and recurrence-free survival curves.** Disease-specific survival curves (**A**, **C**, **E**, **G**) and recurrence-free survival curves (**B**, **D**, **F**, **H**) for all cervical cancer patients included in this study with separate lines for histopathological subtypes (A, B) and for high versus low HLA-E expression shown for the histopathological subtypes separately: cervical squamous cell carcinomas (SCC; C, D), cervical adenosquamous carcinomas (ASC; E, F), and cervical adenocarcinomas (AC; G, H). Disease-specific survival was defined as time (months) from date of primary surgery until death by cervical cancer or date of last follow-up. Recurrence-free survival was defined as time (months) from date of primary surgery until date of first recurrence or date of last follow-up in case of no recurrence. HLA-E expression scores are dichotomized by the median; HLA-E high represents a final expression score ≥6 and HLA-E low represents a final expression score <6. Survival curves are generated by the Kaplan-Meier method and *P* values are calculated by the Log Rank (Mantel-Cox) test.

Univariate and multivariate analyses were performed to determine whether HLA-E expression was associated with disease-specific and recurrence-free survival in cervical cancer patients. Figure 2A and 2B contain the Kaplan-Meier survival curves for disease-specific and recurrence-free survival of all patients included in this study, with separated lines for the histopathological subtypes.

In addition, the Kaplan-Meier survival curves for disease-specific survival (Figures 2C, E, and G) and recurrence-free survival (Figures 2D, F, and H) are shown for high versus low HLA-E expression in SCC (2C, 2D), ASC (2E, 2F), and AC patients (2G, 2H). Neither disease-specific survival nor recurrence-free survival was significantly different for high HLA-E expression versus low HLA-E expression when comparing all histopathological subtypes (*P* = 0.113 and *P* = 0.079, respectively). However, stratifying by histopathological subtype revealed that high HLA-E expression was strongly associated with improved disease-specific and recurrence-free survival in cervical AC (*P* = 0.005 and *P* = 0.001, respectively), but not in cervical SCC or ASC subtype.

Risk was examined in a multivariate Cox regression analysis for disease-specific survival and recurrence-free survival, correcting for age, FIGO stage, histopathological subtype, tumour size, infiltration depth, LVSI, tumour positive resection margins, tumour positive parametrial infiltration, lymph node metastasis and postoperative radiotherapy. HLA-E expression was not significantly associated with disease-specific (hazard ratio (HR) 0.74 (95% CI 0.39-1.42), *P* = 0.368) or recurrence-free survival (HR 0.67 (0.36-1.24), *P* = 0.201). However, after correction, tumour size (HR 1.05 (1.03-1.07), *P* = 0.000) and HR 1.05 (1.03-1.07), *P* = 0.000), tumour positive parametria (HR 2.30 (0.89-5.94), *P* = 0.087 and HR 3.01 (1.21-7.49), *P* = 0.018) and lymph node metastasis (HR 4.31 (2.07-8.95), *P* = 0.000 and HR 3.40 (1.69-6.86), *P* = 0.001) were strong independent predictors for disease-specific and recurrence-free survival. Moreover, the AC histopathological subtype was also a strong and independent predictor for disease-specific (HR 2.79 (1.16-6.70), *P* = 0.022) and recurrence-free survival (HR 3.26 (1.48-7.18), *P* = 0.003).

## Discussion

Over the last few decades, rising incidence rates of cervical AC have been observed relative and absolute to the decreasing incidence rates of cervical SCC, predominantly in younger women and in developed countries [5-9]. Evidence is accumulating that cervical AC is a distinct clinical entity [10,11].

In the present study we have detected high expression of HLA-E in the majority of the three most common histopathological subtypes of cervical cancer. Interestingly, high expression of HLA-E was more frequently observed in cervical AC than in SCC and ASC. High expression of HLA-E in AC was strongly associated with favourable disease-specific and recurrence-free survival. Given the rising incidence rates of cervical AC, it is important to distinguish this histopathological variant from the more common SCC variant.

In most studies concerning cervical cancer, the majority of included tumours are of the SCC subtype, whereas the less-common AC and ASC subtypes comprise only a minority of cases. Moreover, in most studies the AC and ASC cases are combined into one A(S)C subgroup. In this report we compared large and well-defined cohorts of AC and ASC cases with a reference cohort of SCC cases; the SCC and ASC subtypes were accurately distinguished from each other by PAS+/AB staining. Furthermore, the AC subtypes included in this investigation were only the most common and HPV-related AC subtypes: “usual-type” endocervical AC, intestinal-type AC, mucinous AC (not otherwise specified), and endometrioid-type AC [2,4].

Several baseline characteristics distinguished the three histopathological subtypes in this study. In concordance with previous reports, the AC patients and the ASC patients were significantly younger at time of diagnosis than the SCC patients (mean ages 44 and 43 years versus 50 years, respectively) [5,28,29]. Why AC and ASC present more frequently at a younger age is currently unclear. Tumour size, tumour infiltration depth, and LVSI also significantly differed between the histopathological subtypes, with SCC being larger and more invasive than ASC and AC. This observation is important, as it is the size of the tumour, its depth of invasion, and the presence of LVSI that predict prognosis in many cases [4].

The most important prognostic histopathological factors, tumour stage and lymph node status, were equally distributed between the histopathological subgroups in this study. However, selection of patients with tumour-positive lymph nodes revealed a clear trend: the worst recurrence-free survival rates occurred for ASC patients, followed by AC patients and a more favourable recurrence-free survival for SCC patients (*P* = 0.058; data not shown). In addition, multivariate Cox survival analysis indicated that histopathological subtype is an independent prognostic factor for survival, with worse disease-specific and recurrence-free survival for the ASC and AC subtypes (hazard ratios for disease-specific survival, 2.15 and 2.79 respectively; for recurrence-free survival, 2.00 and 3.26, respectively; hazard ratios are relative to SCC).

Cervical cancer is induced by persistent infection with HPV, although different associations with HPV have been described for distinct histopathological subtypes [14,30]. In our study, HPV positivity and the distribution of HPV types also differed significantly among the histopathological subgroups. A high-risk HPV type was detected in 95-97% of all SCC and ASC patients, but only 85% of the AC patients. A possible explanation for the higher rate of HPV-negative cases in AC is that glandular cells support a productive HPV infection to a lesser extent, leading to a low viral load in AC. The detection of a positive high-risk HPV infection in cervical AC can therefore be more difficult compared to SCC and ASC [13,31]. In agreement with the literature, in this study HPV 16 was the most frequently detected type in 68% of the SCC cases, 51% of the ASC cases, and 44% of the AC cases, followed by HPV 18 (17% SCC, 32% ASC, and 44% AC). Only 12% of the AC cases were caused by infections with HPV other than types 16 and 18 (mainly HPV 45), compared to 15% and 16% of the SCC and ASC cases, respectively.

The immune system plays an important role in the progression from initial HPV infection to the development of cervical cancer [17]. The non-classical HLA class Ib molecule HLA-E is involved in the immunological response in various malignant and virally infected cell types [18,20,21]. This study was designed to determine the differences in HLA-E expression in the three most common histopathological subtypes of cervical cancer. High expression of HLA-E was found in 56% of all cases. We confirmed our previous observation of HLA-E expression in SCC [23], and demonstrated high expression of HLA-E in ASC and AC. Furthermore, high expression of HLA-E was more frequently observed in AC than in SCC and ASC (69% vs. 47% and 47%, respectively; *P* = 0.010). We also noted that high expression of HLA-E was associated with negative LVSI (62%, *P* = 0.045). However, the function of high HLA-E expression on cervical tumour cells and on the tumour cells of cervical AC in particular remains uncertain.

Previously, increased expression of HLA-E in several malignant cell types was described in combination with down-regulation of other HLA class I proteins [22,32]. HLA-E function has been investigated in the context of HPV, hepatitis C virus, human cytomegalovirus, human immunodeficiency virus, Epstein-Barr virus, and influenza virus infections [22], leading to the hypothesis that viral peptides are bind to HLA-E, upregulate its surface expression, and lead to the inhibition of NK cells through binding to the CD94/NKG2A receptor. Combined with the decreased surface expression of HLA class I molecules, the infected (tumour) cells gradually lose their capacity for HLA class I peptide presentation to CTLs and subsequently escape from immune attack [24,33-36].

We have previously shown that there are a high number of CTLs in the tumour cell nests and tumour stroma of cervical carcinomas [26]. However, as in most solid tumours, NKs barely infiltrate these tumours [23,37]. Furthermore, tumour-infiltrating CTLs in cervical carcinomas lack expression of the activating receptor CD94/NKG2C, but, in contrast, do express the inhibiting receptor CD94/NKG2A [23,38]. Upregulation of this inhibiting receptor on CTLs can also be induced by the multifunctional cytokine transforming growth factor-beta (TGF-β) [38], which critically contributes to cervical carcinogenesis [39-41]. However, in cervical AC, activation of the TGF-β pathway is more frequently impaired than in SCC [unpublished data]. This observation may imply that the CD94/NKG2A receptor is less abundantly expressed on CTLs in cervical AC, leading to less binding of HLA-E and increased effector function [18,38].

In this study, high HLA-E expression was associated with better long-term disease-specific and recurrence-free survival in the cervical AC subtype. However, this effect disappeared in the multivariate analysis, where only histopathological subtype, tumour size, tumour-positive parametrial infiltration, and tumour-positive lymph nodes were independent predictors for survival and recurrent disease. Studies concerning the prognostic value of HLA-E expression are controversial. Associations between HLA-E expression and improved survival have been described for colorectal carcinoma [42-44], breast carcinoma [45], and glioblastoma [46]. Several studies have reported that HLA-E overexpression is correlated with tumour progression, exhibiting a trend toward worse survival [43-45]. Some reports speculate that high HLA-E expression by malignant cells may represent a selective pro-host advantage, possibly related to a better response rate to subsequent therapies [46]. Benevolo et al. interpret their measurements of HLA-E expression in colorectal carcinoma by the immunoediting model [47], proposing a dual outcome model in which HLA-E is on one hand the first immunological control for HLA-edited tumour variants and on the other hand a trigger for active immune responses [42]. Here, we also suggest that the inhibiting and activating functions of HLA-E may be related to the delicate balance between immune escape and immune surveillance in cervical tumours. However, functional studies will be needed to further unravel the role of HLA-E expression and the underlying mechanisms in cervical cancer and in cervical AC in particular. In most studies, cervical AC comprises only the minority of cases; therefore a multicenter study would be highly valuable to obtain more AC cases.

## Conclusion

In summary, this investigation was the first study of HLA-E expression in a large and well-defined cohort of cervical AC, ASC, and SCC patients. High expression of HLA-E occurred in 53% of all histopathological subtypes. In cervical AC specifically, a significantly higher rate of high HLA-E expression was associated with improved long-term disease-specific and recurrence-free survival. In addition, this study also highlights the importance of careful and reliable histopathological evaluation to precisely define histopathological tumour subtypes. Insight into the biological behaviour and the distinct molecular carcinogenetic processes of the AC, ASC, and SCC subtypes may contribute to the development of tumour-specific treatment strategies, future vaccine development, and the design of tumour-specific immunotherapies.

## Abbreviations

AC: Cervical adenocarcinoma; ASC: Cervical adenosquamous carcinoma; CTLs: Cytotoxic T lymphocytes; FIGO: International Federation of Gynaecology and Obstetrics; HLA: Human leukocyte antigen; HPV: Human papillomavirus; LVSI: Lymph-vascular space invasion; NKs: Natural killer cells; SCC: Cervical squamous cell carcinoma; TMA: Tissue microarray.

## Competing interests

The authors declare that they have no competing interests.

## Authors’ contributions

This research was designed by ESJ and GJF. VMS collected the data and AAWP contributed to the acquisition of data by supervising patient follow-up. VMS and GJF reviewed all tumours and VMS and ESJ scored the immunohistochemistry assays. VMS and ESJ analysed all data and drafted the paper, which was critically revised by GJF and AAWP. All authors read and approved the final manuscript.
